# Food intake profiles of children aged 12, 24 and 48 months from the 2004 Pelotas (Brazil) birth cohort: an exploratory analysis using principal components

**DOI:** 10.1186/1479-5868-9-43

**Published:** 2012-04-17

**Authors:** Giovanna Gatica, Aluisio J D Barros, Samanta Madruga, Alicia Matijasevich, Iná S Santos

**Affiliations:** 1 Postgraduate Program in Epidemiology, Federal University of Pelotas, Pelotas, Brazil

## Abstract

**Objectives:**

To identify food intake profiles of children during their first four years of life and assess its variations according to sociodemographic and behavioral characteristics.

**Methods:**

The Pelotas Birth Cohort Study (Brazil) recruited 4,231 liveborns, who were followed-up at ages 3, 12, 24 and 48 months. Food consumption data of children aged 12, 24 and 48 months was collected using a list of foods consumed during a 24-hour period prior to the interview. The food profiles were identified with the use of principal component analysis (PCA) for each age studied.

**Results:**

Five components were identified at each age, four of them similar in all time points, namely: beverages, milks, staple, and snacks. A meat & vegetables component was identified at 12 and 24 months and a treats component at 48 months. The greatest nutritional differences were found among children from different socioeconomic levels. With regard to the milks component, higher breast milk intake compared to cow's milk was seen among poorer children (12- and 24-month old) and higher milk and chocolate powdered milk drink consumption was seen among more affluent children aged 48 months. Poorer children of less educated mothers showed higher adherence to the treats component (48 months). Regarding to the snack component, poorer children consumed more coffee, bread/cookies while more affluent children consumed proportionately more fruits, yogurt and soft drinks. Child care outside of the home was also a factor influencing food profiles more aligned with a healthier diet.

**Conclusions:**

The study results showed that very early in life children show food profiles that are strongly associated with social (maternal schooling, socioeconomic position and child care) and behavioral characteristics (breast-feeding duration, bottle-feeding and pacifier use).

## Introduction

Since the beginning of life, the family, particularly the mother, influences its children’s food preferences [1-3]. Socioeconomic, nutritional and cultural factors also contribute to food preferences [3-7] and these preferences become the eating habits of adolescents and adults [8].

Today there is great interest in nutrition and lifestyles and their effects due to growing rates of obesity and non-transmissible chronic diseases [9,10]. The 2008–2009 Brazilian Family Budget Survey data showed that, from the age of five, there is a rapid increase in the prevalence of overweight and obesity. Excess weight in people increased by over one percent point a year in the last six years, suggesting that in 10 years two-thirds of Brazilian adults will have excess weight, similar to the current US situation [11].

The current literature shows a growing number of studies on food intake patterns based on principal component analysis (PCA) as an alternative approach to the assessment of food consumption and nutrient intake [12-14]. These food patterns reflect the majority of the variation in food consumption in a given population. PCA and similar techniques work reducing the large amount of information collected through food frequency questionnaires to a few factors or components (4 to 6 in most cases). This approach makes it easy to identify complex patterns, as well as the correlation between foods or nutrients [15]. There are many studies in the literature describing food patterns and their role as risk factors for non-transmissible chronic diseases [13] but far less studies have assessed food intake in children.

The present study aimed to identify the food intake profiles of children belonging to the 2004 Pelotas birth cohort study when aged 12, 24 and 48 months using PCA. We chose to use the term *profile* rather than *pattern* because food consumption data were not obtained from food frequency questionnaires (FFQ), the most widely used instrument in food pattern studies. In addition to creating the food profiles, we assessed their variation according to sociodemographic and behavioral characteristics.

## Methods

A birth cohort was started in the city of Pelotas, southern Brazil, in 2004, when 4,231 children were recruited, representing 99.2% of all births to women living in the city’s urban area that year. All eligible newborns were evaluated and their mothers interviewed within 24 hours after birth. A structured questionnaire consisting of nine sections was administered by trained interviewers and information about the family, the mother, the child and aspects related to the pregnancy was collected. The cohort children were followed up when aged 3, 12, 24 and 48 months. They all underwent anthropometric assessments and information about health status, nutrition, child development, housing conditions and socioeconomic position was collected. The follow-up rates of children at 3, 12, 24 and 48 months were 95.7%, 94.2%, 93.4% and 91.8%, respectively. Details on the perinatal and follow-up studies are available elsewhere [16,17].

We used information on sociodemographic and behavioral characteristics from the 2004 perinatal study data. Food consumption was assessed at ages 12, 24 and 48 months. Information on the introduction of complementary food was obtained at ages 3 and 12 months. Food consumption was assessed using a list of food items or food groups consumed in the 24 hours of the last day previous to the interview that the child ate as usual. With this instrument, the number of times each food item was consumed within 24 hours was recorded, but not the amount consumed. The mother was asked whether each food item in the list had been consumed in each of seven meals or periods of the day – wake-up time, morning, lunch, afternoon, dinner, evening, night. Thus, each food item frequency can vary from zero to seven. This approach was developed and applied in a growth curve study carried out in Pelotas from 1997 to 2000 [18] because there was not enough time during the interviews to collect information using an open questionnaire (24-hour food recall) or a FFQ.

The food items on the list for children aged 12 and 24 months were as follows: breast milk, cow’s milk, milk powder, coffee, water or tea, juice, bread/cookies, yogurt, fruits, eggs, rice, beans, vegetables/legumes, pasta, potato or cassava, meat and powdered chocolate milk drinks. The respondents were allowed to report up to two other food items consumed that were not included in the list. Fresh cow’s milk and milk powder were grouped into a single item. The food list for children aged 48 months contained the same items plus soft drinks. The frequency of habitual consumption of chips, chocolate, candies, lollipops and chewing gums – the latter three grouped into a single variable “sweets” – was also assessed and then converted into times a day so that it would be comparable to other foods.

Food profiles for each age studied were identified with the use of PCA followed by varimax rotation to improve component interpretation. The number of components retained was chosen based on the screeplots and on their interpretability. Component scores were calculated for each child in the sample and multiplied by 100 to make their interpretation easier in the tables. For the analysis at 12 and 24 months the 16 food items listed above were used in the PCA. For the analysis at 48 months 17 food items of the 24-hour consumption list and three food items of the habitual consumption list (chips, chocolate and sweets converted into times a day) were used in the PCA.

Following the identification of food profiles for each age, mean component scores for groups of sociodemographic and behavioral characteristics were compared through analysis of variance (ANOVA). These variables included: having siblings (yes/no); maternal age (years); bottle-feeding (at 3 and 12 months of age); total breastfeeding duration (months); age starting pacifier use (months); early (before the age of 3 months) introduction of solid foods (yes/no); national wealth index (quintiles of the Brazilian National Economic Indicator, IEN [19]); maternal schooling (years); and full- or part-time child care outside of the home. Information on breastfeeding and use of pacifiers was obtained from the perinatal study and follow-ups at 3 and 12 months. Child care outside of the home was assessed at 12, 24 and 48 months. Because many variables are strongly associated with socioeconomic position, a crude analysis and an analysis adjusted for wealth quintiles were performed. All analyses were carried out using the statistical package Stata 9.0 (Stata Corp., College Station, TX, USA, 2007).

The mothers of the birth cohort were provided with detailed explanations of the study objectives and procedures and gave their written consent for their children to participate. Confidentiality of information was warranted. Participation in the study was voluntary and they could withdraw from participation at any time without giving reasons. All the study phases were approved by the Research Ethics Committee of the Universidade Federal de Pelotas School of Medicine.

## Results

The first five PCA components for each age studied were retained, as the scree plots showed a clear drop after the fifth component in the three analyses (data not shown). We considered representative of each component the food items that showed a loading greater than 0.3 or less than –0.3. Although all foods were used to calculate component scores, only the representative ones are shown in the results. Complete tables with all food items and respective loadings and standard errors are presented in an Additional file 1: Annex to this article.

For children aged 12 months the first component, called *milks*, included breast milk with a positive loading and cow’s milk or formula with a negative loading. The second component, called *staple*, included staple foods of the Brazilian diet including rice and beans with a positive loading and pasta with a negative loading. The third component, called *meat & vegetables*, included meat, vegetables/legumes and potato/cassava, all with positive loadings. The fourth component, called *beverages*, included juices with a positive loading and water/tea with a negative loading. The fifth component, called *snack*, included coffee and bread/cookies with positive loadings and fruits with a negative loading (Table 1).

**Table 1:** Food intake profiles of children aged 12, 24 and 48 months from the Pelotas birth cohort, 2004

**FOOD PROFILE**	**12 months** **Food item**	**Loading**	**% var**	**24 months** **Food item**	**Loading**	**% var**	**48 months** **Food item**	**Loading**	**% var**
Milks	Breast milk	0.68	11.2	Breast milk	0.65	9.5	Cow’s milk	0.64	9.2
	Cow’s milk	–0.70		Cow’s milk	–0.69		Chocolate milk	0.62	
Staple	Rice	0.65	11.1	Rice	0.67	10.8	Rice	0.62	9.4
	Beans	0.55		Beans	0.61		Beans	0.50	
	Pasta	–0.40		Pasta	–0.38		Meat	0.42	
Meat & vegetables	Meat	0.68	9.1	Meat	0.56	8.0			
	Vegetables/legumes	0.50		Vegetables/legumes	0.54				
	Potato/cassava	0.36		Potato/cassava	0.38				
				Fruits	0.46				
Beverages	Juice	0.66	8.6	Juice	0.68	8.4	Juice	0.71	7.2
	Water/tea	–0.72		Water/tea	–0.71		Soft drinks	–0.52	
Snack	Coffee	0.53	8.4	Coffee	0.58	9.1	Coffee	0.46	7.6
	Bread/cookies	0.63		Bread/cookies	0.58		Bread/cookies	0.35	
	Fruits	–0.38		Yogurt	–0.45		Water/tea	0.34	
							Yogurt	–0.42	
							Soft drinks	–0.39	
Treats							Chips	0.58	7.1
							Sweets	0.57	
							Chocolate	0.43	
Total			48.4			45.8			40.5

For children aged 24 months the *milks*, *staple*, and *beverages* components were also found (Table 1). They included the same food items with similar loadings to those at 12 months. The *meat & vegetables* and *snack* components at this age were slightly different compared to 12 months. The *snack* component included coffee and bread/cookies with positive loadings but yogurt (with a negative loading) instead of fruits. The *meat & vegetables* component, in addition to the food items described at 12 months, also included fruits, with a positive loading.

For children aged 48 months the results differed from the previous ages (Table 1). Again, five components were retained but *meat & vegetables* disappeared, replaced with a new one, called *treats*. The *milks* component included cow’s milk or formula and powdered chocolate milk drinks, both with positive loadings. The *staple* component included rice, beans and meat, all with positive loadings. The *snack* component included coffee, water/tea and bread/cookies with positive loadings, and yogurt and soft drinks with negative loadings. The *beverages* component included juices with a positive loading and soft drinks with a negative loading. The new *treats* component included chips, sweets and chocolate, all with positive loadings. The *beverages* component score at 12 months and the *snack* component score at 48 months had signals for the main food items that were opposite to those in the other ages. Since the sign (direction) of the components is arbitrary, the components were multiplied by –1 to make it easier to compare these same components at different ages.

Components where all loadings are of the same sign are easier to interpret. High scores mean increased adherence or increased consumption of food items representative of the component. Components whose loadings present opposite signs are more complex and suggest contrasting food items. For example, in the *staple* component, rice and beans have a positive loading while pasta has a negative one. High scores in the *staple* component would indicate that the individuals analyzed consume proportionally more rice and beans than pasta while low (negative) scores would indicate they consume proportionately more pasta. However, it does not mean greater absolute pasta consumption compared to the other food items.

Tables 2, 3 and 4 show mean component scores at 12, 24 and 48 months of age according to independent variables. There were differences between the groups for almost all components. The variable showing the greatest differences was undoubtedly socioeconomic position.

**Table 2:** Mean component scores of food intake profiles at 12 months according to explanatory variables. Pelotas, Brazil, 2005

**Variable**	**Milk** **Mean**	**Staple** **Mean**	**Meat & vegetables** **Mean**	**Beverages** **Mean**	**Snack** **Mean**
Sibling living at home	0.1	<0.001	<0.001	0.002	<0.001
No	–3.73	–9.29	15.52	6.23	–13.25
Yes	3.32	8.26	–13.81	–5.54	11.79
Gender	0.007	0.5	0.8	0.6	0.01
Male	–5.58	–1.40	0.47	0.98	–4.66
Female	6.03	1.51	–0.50	–1.06	5.04
Maternal age at birth (years)	0.2	0.009 (0.5)*	<0.001	0.02	<0.001 (0.3)*
<20	–8.44	7.46	–16.27	6.21	13.82
20–29	0.13	3.78	–1.90	2.54	1.54
30–39	4.66	–11.17	15.18	–5.45	–11.24
40 or more	5.44	–2.64	–8.21	–23.88	–8.12
Wealth quintiles (IEN)	0.01	<0.001	<0.001	<0.001	<0.001
1^st^ (poorest)	5.02	24.69	–44.84	–19.47	38.25
2^nd^	–1.38	13.80	–9.91	5.24	8.06
3^rd^	–1.46	1.31	4.11	6.71	–5.12
4^th^	11.07	–12.82	19.38	6.93	–17.21
5^th^ (richest)	–13.91	–38.84	47.30	5.36	–37.75
Maternal schooling (complete years)	0.2	<0.001	<0.001	<0.001 (0.05)*	<0.001
0–3	10.40	28.97	–48.48	–22.23	34.85
4–7	–2.13	21.08	–32.22	–4.36	24.63
8–10	4.27	–0.62	1.20	6.95	–5.04
11 or more	–4.35	–27.47	41.27	4.54	–27.47
Care outside the home	<0.001	0.04 (0.2)*	0.001 (0.1)*	0.9	<0.001
No	2.33	1.08	–1.55	–0.09	1.78
Yes	–38.62	–18.11	26.43	1.44	–29.97
Bottle-feeding	<0.001	0.07	<0.001	0.002	<0.001
At 3 and 12 months of age	–41.51	4.10	–4.30	4.33	1.75
At 3 months but not at 12 months of age	176.41	2.07	–35.53	–17.10	39.32
Not at 3 months but at 12 months of age	32.78	–3.88	15.03	–3.37	–13.04
Never	195.84	–17.26	–0.85	–21.25	13.40
Breast-feeding duration	<0.001	0.2	0.7	0.09	1.0
Up to 7 days	–83.44	7.19	3.26	–3.02	–1.55
8 days |–3 moths	–87.56	7.09	–1.12	7.62	0.14
3 |– 6 months	–84.16	–5.57	–4.33	4.07	–1.06
6 months or more	68.51	–1.47	1.50	–3.80	0.45
Age started pacifier use (months)	<0.001	0.2	<0.001	0.1	0.05
< 3 months	–52.10	3.39	–4.03	2.74	2.29
3 months or more	–41.94	–8.01	19.34	3.55	–11.21
Never	94.70	–1.72	–1.95	–5.36	1.08
Early introduction of solid foods (< 3 mo.-old)	<0.001	<0.001	<0.001 (0.05)*	0.5	<0.001
No	4.88	–3.96	3.35	0.52	–5.20
Yes	–30.46	24.73	–20.96	–3.27	32.46

* *P*-values adjusted for wealth (IEN) were presented only when there was a loss of statistical significance after adjustment. IEN: indicador econômico nacional, or national wealth index.

(N = 3,827)

**Table 3:** Mean component scores of food intake profiles at 24 months according to explanatory variables. Pelotas, Brazil, 2006

**Variable**	**Staple** **Mean**	**Milk** **Mean**	**Snack** **Mean**	**Beverage** **Mean**	**Meat & vegetables** **Mean**
Sibling living at home	0.05	0.01	<0.001	0.06	<0.001
No	–4.47	–5.39	–21.27	3.70	8.72
Yes	4.02	4.86	18.98	–3.32	–7.80
Gender	0.3	0.03	1.0	0.06	0.5
Male	2.14	–4.26	0.01	3.42	–1.16
Female	–2.29	4.68	–0.02	–3.74	1.25
Maternal age at birth (years)	0.002 (0.06)*	0.01	0.08	0.2	<0.001 (0.05)*
<20	15.11	–10.22	5.34	–5.05	–14.23
20–29	–1.52	–1.40	2.50	3.85	0.02
30–39	–4.61	7.48	–7.65	–2.89	10.30
40 or more	–24.67	13.96	–4.60	–4.72	–5.22
Wealth quintiles (IEN)	<0.001	0.005	<0.001	<0.001	<0.001
1^st^ (poorest)	28.19	7.92	53.80	–17.23	–28.38
2^nd^	2.23	6.06	7.61	3.74	–2.40
3^rd^	2.88	–6.23	–9.85	5.72	8.02
4^th^	–11.14	3.46	–20.17	2.21	9.30
5^th^ (richest)	–32.46	–12.28	–49.11	8.80	20.14
Maternal schooling(complete years)	<0.001	0.006 (0.06)*	<0.001	0.007 (0.5)*	<0.001
0–3	23.18	18.86	48.90	–14.04	–35.72
4–7	17.40	4.17	29.99	–4.34	–19.91
8–10	1.98	–6.37	–2.89	–0.33	6.95
11 or more	–25.51	–4.09	–39.48	8.05	23.02
Care outside the home	0.2	0.005	<0.001	<0.001	<0.001
No	1.07	2.27	6.34	–2.73	–3.34
Yes	–6.58	–13.92	–38.86	16.74	20.45
Bottle-feeding	0.5	<0.001	<0.001	0.01	<0.001
At 3 and 12 months of age	1.93	–25.79	1.09	3.61	–5.84
At 3 months but not at 12 months of age	–6.19	134.25	39.67	–4.40	–9.68
Not at 3 months but at 12 months of age	–4.79	16.30	–11.36	–4.20	10.14
Never	2.50	130.14	11.09	–20.28	22.08
Breast-feeding duration	0.1	<0.001	0.5	0.002	<0.001
Up to 7 days	20.20	–42.35	–6.92	17.28	–10.19
8 days |–3 moths	–3.51	–51.88	–1.88	10.11	–13.25
3 |– 6 months	–2.39	–48.06	–3.92	0.61	1.32
6 months or more	0.14	38.19	2.59	–5.51	5.32
Age started pacifier use (months)	0.3	<0.001	0.06 (0.03)*	<0.001	0.003
< 3 months	2.94	–32.54	–1.40	4.87	–4.71
3 months or more	–4.62	–22.86	–8.60	7.28	14.30
Never	–2.97	59.60	5.60	–10.27	1.35
Early introduction of solid foods (< 3 mo.-old)	<0.001	0.04	<0.001	0.3	0.006 (0.2)*
No	–3.52	2.07	–4.77	–0.98	2.07
Yes	20.47	–10.05	29.43	5.13	–12.64

* *P*-values adjusted for wealth (IEN) were presented only when there was a loss of statistical significance after adjustment. *IEN*: indicador econômico nacional, or national wealth index.

(N = 3,790).

**Table 4:** Mean component scores of food intake profiles at 48 months according to explanatory variables. Pelotas, Brazil, 2008

**Variable**	**Staple** **Mean**	**Milk** **Mean**	**Snack** **Mean**	**Beverage** **Mean**	**Treats** **Mean**
Sibling living at home	0.004 (0.09)*	<0.001	<0.001	0.04 (0.1)*	0.007 (0.1)*
No	–6.88	10.91	–24.79	4.28	–5.63
Yes	6.09	–9.65	21.92	–3.78	4.98
Gender	0.2	0.6	0.2	0.003	0.03 (0.05)*
Male	2.54	1.04	–2.37	5.64	–4.02
Female	–2.76	–1.13	2.57	–6.12	4.36
Maternal age at birth (years)	0.003 (0.07)*	0.03 (0.3)*	0.2	0.01 (0.08)*	<0.001
<20	14.02	–12.03	9.46	–8.83	18.96
20–29	–1.43	0.79	–1.84	–0.77	2.55
30–39	–3.61	4.40	–3.05	8.97	–16.50
40 or more	–28.70	19.49	–2.39	–12.65	–6.27
Wealth quintiles (IEN)	<0.001	<0.001	<0.001	0.005	<0.001
1^st^ (poorest)	26.23	–43.03	57.68	–4.96	22.48
2^nd^	11.68	–9.40	14.46	–4.89	10.63
3^rd^	3.45	16.29	–10.80	–5.81	3.70
4^th^	–17.46	23.43	–31.68	4.72	–17.25
5^th^ (richest)	–36.74	26.80	–51.03	14.31	–29.63
Maternal schooling (complete years)	<0.001	<0.001	<0.001	<0.001	<0.001
0–3	17.63	–53.46	81.55	–16.99	21.93
4–7	17.51	–23.19	29.02	–6.57	30.04
8–10	2.23	18.41	–5.35	–1.77	–6.42
11 or more	–24.35	22.58	–44.14	10.46	–30.12
Care outside the home	<0.001	<0.001	<0.001	<0.001	<0.001
No	11.16	–10.24	19.37	–10.54	12.10
Yes	–17.42	16.00	–30.24	16.46	–19.00
Bottle-feeding	0.6	<0.001	0.02 (0.7)*	0.7	0.001 (0.06)*
At 3 and 12 months of age	1.37	5.83	1.58	0.52	4.71
At 3 months but not at 12 months of age	1.40	–70.64	20.45	–9.16	22.42
Not at 3 months but at 12 months of age	–4.40	6.32	–10.54	3.88	–10.58
Never	6.22	–42.00	2.83	–1.14	–7.02
Breast-feeding duration	0.02	<0.001	0.4	0.2	0.1
Up to 7 days	8.41	–7.52	–9.84	–15.58	10.61
8 days |–3 moths	9.22	14.91	4.87	3.22	7.00
3 |– 6 months	–12.07	10.41	–4.15	–2.60	–3.11
6 months or more	–0.43	–7.79	0.18	1.38	–2.53
Age started pacifier use (months)	0.5	<0.001	0.3	0.7	0.2
< 3 months	2.24	0.97	0.96	1.65	0.29
3 months or more	–3.56	18.97	–8.87	–3.27	–7.78
Never	–2.92	–8.92	0.58	1.18	3.57
Early introduction of solid foods (< 3 mo.-old)	0.04 (0.6)*	0.3	<0.001	0.6	<0.001
No	–2.45	1.13	–4.94	1.16	–3.39
Yes	10.80	–5.21	26.17	–1.76	23.26

* *P*-values adjusted for wealth (IEN) were presented only when there was a loss of statistical significance after adjustment. *IEN*: indicador econômico nacional, or national wealth index.

(N = 3,714).

**Figure 1:**
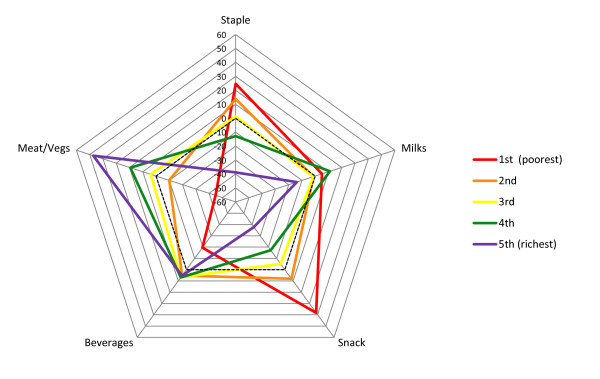
Food intake profiles of children aged 12 months according to socioeconomic quintiles.

**Figure 2:**
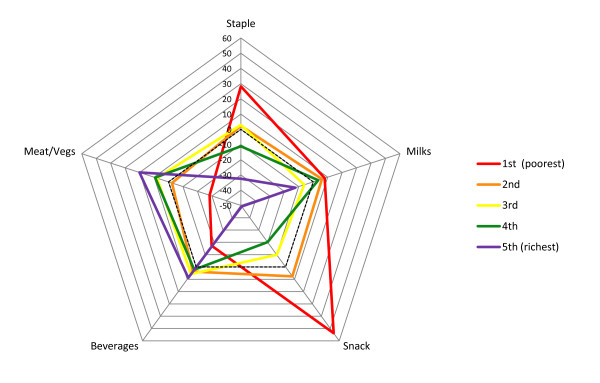
Food intake profiles of children aged 24 months according to socioeconomic quintiles.

**Figure 3:**
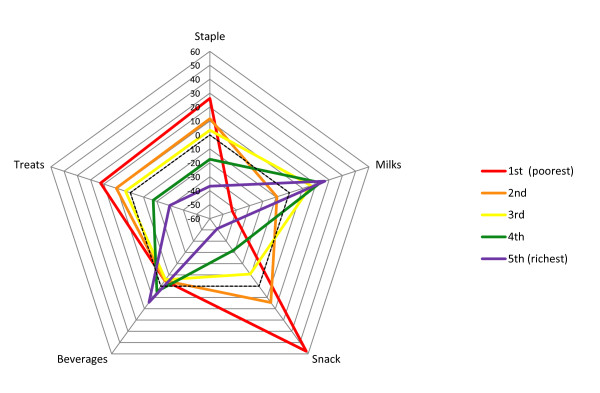
Food intake profiles of children aged 48 months according to socioeconomic quintiles.

*Milks* component – At 12 months the *milks* component presented differences across wealth quintiles, but not a clear trend. The fourth wealth quintile showed a higher score compared with the other groups, which may be due to the fact that it had the highest number of breastfed children. At 24 months a pattern similar to that at 12 months was seen but with minor differences between the quintiles (Tables 2 and 3; Figures 1 and 2). At 48 months, when breast milk is no longer present, there was a monotonic trend of increasing *milks* component scores according to socioeconomic position, showing greater consumption of milk and powdered chocolate milk drinks among better-off families (Figure 3). Similarly, there was a positive association between the *milks* component and maternal schooling.

*Staple* component – At 12 months, the poorer the family the higher the *staple* score, showing proportionately greater consumption of rice and beans compared to pasta (Table 2, Figure 1). A similar trend was seen at 24 months (Table 3, Figure 2). Maternal schooling presented analogous associations. At 12 and 24 months, children who were fed with solid foods at an early age showed a proportionally greater consumption of rice and beans than pasta. At 48 months, the *staple* component showed decreasing scores with increasing socioeconomic position, showing lower consumption of rice, beans and meat among more affluent children compared to poorer ones (Figure 3). The same was seen when comparing children of high- and low-schooling mothers. Among children aged 48 months cared outside of the home the consumption of rice, beans and meat was lower.

*Snack* component – At 12 and 24 months, the poorer the family the higher the *snack* score, showing proportionately higher consumption of coffee and bread/cookies than fruits and yogurt at 12 and 24 months, respectively (Tables 2 and 3; Figures 1 and 2). A similar trend was observed according to maternal schooling. Also, having a sibling living in the same house, being cared at home, receiving solid foods at an early age and bottle-feeding at 3 and 12 months of age, at 3 months of age only or never were all associated with proportionately greater consumption of coffee and bread/cookies than fruits and yogurt at 12 and 24 months. Children who had never used a pacifier by the age of 24 months had a proportionately higher consumption of coffee and bread/cookies than yogurt. At 48 months, the *snack* component scores tended to decrease with increasing socioeconomic position, showing greater proportional consumption of coffee, water/tea and bread/cookies than yogurt and soft drinks among those at the lower strata compared to those at the upper strata (Table 4, Figure 3). A similar trend was seen for maternal schooling. Among children who had siblings living in the same house, who were cared for at home and received solid foods at an early age, the consumption of coffee, water/tea, bread/cookies was proportionally greater than that of yogurt and soft drinks.

*Beverage* component – At 12 and 24 months, there was a positive association of the *beverage* component and socioeconomic position. A negative association with maternal age was seen only at 12 months. Twelve-month-old children who had siblings living in the same house and had never been or were not bottle-fed at 12 months of age showed lower scores, showing proportionately greater consumption of water/tea than juice. At 24 months, children cared for at home, who were never bottle-fed, never used pacifiers and were breast-fed for six months or more had lower scores. At 48 months, there was a positive association with socioeconomic position and maternal schooling. Lower scores showing a proportionally greater consumption of soft drinks than juice were seen among children who were cared for at home.

*Meat & vegetables* component – At 12 and 24 months, there was a positive association of the *meat & vegetables* component with socioeconomic position and maternal schooling. Twelve-month-old children who had no siblings living in the same house, their mothers were 30 to 39 years of age, were only bottle-fed at 12 months of age and used pacifiers when they were 3 months old or more had higher scores indicating greater adherence to this component, i.e., greater consumption of meat, vegetables/legumes and potato/cassava. Twenty-four-month-old children who had no siblings living in the same house, were cared for outside of the home, were only bottle-fed at 12 months of age or at 3 and 12 months of age and started using pacifiers at the age of 3 months or never used a pacifier had higher scores.

*Treats* component – The higher consumption of sweets, chocolate and crisps (only available at 48 months) was negatively associated with socioeconomic position and maternal schooling. In addition, children of mothers younger than 30, cared for at home and who received solid foods at an early age had higher scores showing greater consumption of these food items.

## Discussion

The present study identified the food profiles of children from a birth cohort when aged 12, 24 and 48 months. We have identified dietary profiles that closely reflect the way children are fed in the study area. More importantly, in a period of life where diet is often monotonous [20] we have documented marked differences across groups related to socioeconomic and cultural characteristics. The wealth classification we used (based on quintiles of a household asset index) presented the strongest association with the food profiles, showing that, from early age, children from different economic levels eat in rather different ways. Although our analysis did not focus on the quality of the diet, richer children at one year of age ate more meat and vegetables, fruit and juices, suggesting a better and more varied diet. At 4 years of age, the poorer children were eating more chips, sweets and chocolate. These early differences are very important since they are likely to shape future diet, since children get to like what they eat more, and usually eat only what they like [21].

Several other factors were strongly associated with the food profiles – presence of siblings, gender, day care, breastfeeding duration, age at introduction of solid foods. If the theory that early life exposure is capable to shape future diet [21] is correct, then all these characteristics are relevant to the individual’s diet from birth.

It is noteworthy the low loss rate of this cohort at all ages studied (<10%) and the regular frequency of data collection on food consumption, minimizing recall bias. In addition, following up all children born in a given year in the same city makes the sample representative of the source population. One limitation of the study is the source of food consumption information. The 24 hours preceding the interview is a short period of time for assessing food consumption and does not reflect food habits of children. It does not allow to establishing food patterns that could be predictors of other outcomes, either. However, Robinson et al. [15] examined the first three components defined by PCA from a 24-hour food recall for children aged 6 months and found that the results were very similar to those obtained using the FFQ. This finding supports that the results are valid for groups, despite not being suitable for use as individual risk indicators given the variability of the food consumption of a single day in comparison to the dietary habit.

We found in this study components that reflect the major food groups consumed during childhood, which are consistent with the most common feeding practices during childhood including milk, bread-based snacks, cookies, fruits, juices and teas, and the preference for rice, beans and pasta as staple foods as reported by other authors [22,23]. At 12 and 24 months, the components reflect weaning practices. They show the importance of breast milk during the first year of life and the decline in its importance in children’s diets until it is completely substituted by the age of 48 months. Consistent results and components with positive and negative loadings were also reported in a British study in 2007 that investigated eating patterns of children aged 6 and 12 months. In that study, in the second component at 6 months, breast milk had a negative score and adult foods had a positive one clearly showing a contrast (negative correlation) between the consumption of breast milk and solid foods and cow’s milk. At 12 months, breast milk was no longer seen in any component [15].

The present study showed that at 12 months children in the fourth socioeconomic quintile had a proportionately higher consumption of breast milk than those in the first quintile. This same pattern was still detectable at 24 months, though breastfeeding was less frequent. Horta et al. [24] using data from two birth cohorts from the same location of the present study reported that from the age of 9 months breast-feeding was more prevalent among poorer than more affluent children while the contrary was seen in the first months of life. Faleiros et al. [25] argue that in developed countries poor children are breastfed for shorter periods of time because less privileged, less educated mothers have less access to information about the benefits of breastfeeding. This same feeding pattern is seen in more developed regions of Brazil, i.e., more educated women with higher socioeconomic position breast-feed their children for longer periods of time [25-27]. Overall, no clear picture emerges as the country is going through important changes in breastfeeding behavior and there are several cultural and economic forces acting over it.

Each population has a set of foods, typical of the country or region, that are frequently consumed, usually due to cultural aspects and availability [28]. In Brazil, rice and beans are common staple foods accompanied by meat, green vegetables and cassava flour. Romaguera et al. [29] studied children and adolescents in the Andean region of Argentina and found two components; one of them, called *autochthonous Andean*, included typical foods of that region. In the UK, in 2005 [12] and 2008 [14], Northstone et al. found at all ages studied in the ALSPAC (3, 4, 7 and 9 years) a component they called *traditional* that included typical foods in this population: meat, tubers, green vegetables, peas and corn. This component was very similar to the *meat & vegetables* component identified among children of Pelotas. Foods with positive loadings in the *staple* component at 12 and 48 months were consistent with those described in other studies in Brazilian adults [30,31]. At 48 months, the *meat & vegetables* component was no longer present and the *staple* component included, in addition to rice and beans, meat (with a positive loading). This change is clearly related to the way foods are combined in children’s meals at different ages, meat being served with rice and beans more often for older children.

Because of the original design of the instrument used, our food list did not include processed foods such as meat products, frozen or canned foods, sauces, candies, chewing gum, etc. Thus, we could not identify components specifically associated to a profile of processed foods that include foods with low nutritional value as reported in other studies [12,14,32,33]. However, chocolate, candies and chips – found in the components identified in the above mentioned studies – were also found in the present study in the *treats* component and soft drinks in the *beverages* and *snack* components at 48 months, in a complementary survey to the food list used. At 12 and 24 months some mothers reported the consumption of these food products together with “other foods” consumed by their children. However, these foods were not included in the analysis because they involved a wide variety of items in an inconsistent manner (data not shown). In France, Lioret et al. [34] found a pattern, *snack* and *sedentary*, characterized by the consumption of potato chips, processed juice, soft drinks and low levels of physical activity with positive loadings. Yogurt/cottage cheese, ham/honey, and water had all negative loadings. The *snack* component of children in Pelotas includes both foods with high and low nutritional value while the *snack* component of these other studies included only foods with low nutritional value.

Several studies [12,32,33,35] found a positive association between the component containing processed foods, chips, soft drinks, etc. and being a child of a mother with low schooling and low socioeconomic position, having older siblings and early introduction of solid foods, after adjusting for each other. These results are consistent with the associations found for foods with low nutritional value of the *treats* component and soft drinks of the *snack* and *beverage* components at 48 months.

The *healthy* and *traditional* components of these studies [12,32,33,35] were associated with more educated mothers as well as less time spent watching TV a day, high socioeconomic position, mothers living with a partner, older and vegetarian mothers. A comparison with these studies was not possible since these components did not include the same foods of the *staple* and *meat & vegetables* components in our study.

Our findings regarding breastfeeding are corroborated by other studies showing higher consumption of breast milk when it was not associated with bottle-feeding and pacifier use [36,37] and early introduction of solid foods [38].

## Conclusions

The present study not only identified the components that summarize food intake habits of children in south/southeast Brazil but also showed that the food profiles are associated with behavioral (breast-feeding, bottle-feeding and pacifier use) and socioeconomic variables (wealth and schooling) as well. Although these results are not surprising, our study empirically confirmed there are dietary differences between social groups from the very beginning of life. Marked differences between groups were seen even in a phase of characteristically “monotonous” dietary patterns [22], as clearly shown in the graphs. These distinct practices from an early age will likely determine different eating patterns in adulthood [39,40] and, consequently, different risk profiles. Northstone et al. [14] claim children should be regularly evaluated during childhood to accurately assess dietary effects on future outcomes. Further studies on dietary patterns in children are thus needed, taking into account cultural and regional differences. This knowledge can help assessing short- and long-term dietary effects and develop interventions for health promotion.

## Abbreviations

PCA = Principal component analysis; FFQ = Food Frequency Questionnaire; IEN = National wealth index; ALSPAC = The Avon Longitudinal Study of Pregnancy and Childhood.

## Conflict of interests

The authors declare no conflict of interests.

## Author contributions

This study was conducted by GLMG, AJDB and SWM. GLMG performed the analyses and drafted the manuscript. AB proposed the idea, supervised the analyses and helped draft the manuscript. SWM helped in some analyses and contributed to the nutritional focus of the study. ISS and AM participated in the design and conduct of the original cohort study as well as in interpreting results and reviewing the manuscript. All authors read and approved the final manuscript. The study is based on GLMG master dissertation supervised by AJDB.

## Supplementary Material

Additional file 1 Full food profiles for children 12, 24 and 48 months of age Click here for file
