# Supplementary material for: Food intake profiles of children aged 12, 24 and 48 months from the 2004 Pelotas (Brazil) birth cohort: an exploratory analysis using principal components
Source: Int J Behav Nutr Phys Act. 2012 Apr 17;9:43. doi: 10.1186/1479-5868-9-43 (PMC3424118; doi:10.1186/1479-5868-9-43)
Supplement: Additional file 1 — Full food profiles for children 12, 24 and 48 months of age [file 1479-5868-9-43-S1.doc]

# Food profiles at 12 months of age

Table 1 – Sample size, mean, standard deviation, minimum and maximum for the frequency of consumption in 24 hours of each food item used for the profiles of children age 12 months.

| **Food item** | **N** | **Mean** | **s.d.** | **Min.** | **Max.** |
| --- | --- | --- | --- | --- | --- |
| **Breast milk** | 3827 | 1.35 | 1.92 | 0 | 7 |
| **Cow’s milk** | 3827 | 2.52 | 1.57 | 0 | 7 |
| **Coffee** | 3827 | 0.08 | 0.33 | 0 | 5 |
| **Water/Tea** | 3827 | 0.63 | 0.90 | 0 | 5 |
| **Juice** | 3827 | 1.15 | 1.13 | 0 | 5 |
| **Bread/Cookie** | 3827 | 1.20 | 0.84 | 0 | 4 |
| **Yogurt** | 3827 | 0.75 | 0.78 | 0 | 5 |
| **Fruit** | 3827 | 0.90 | 0.80 | 0 | 4 |
| **Egg** | 3827 | 0.08 | 0.29 | 0 | 2 |
| **Rice** | 3827 | 1.20 | 0.80 | 0 | 3 |
| **Beans** | 3827 | 1.26 | 0.78 | 0 | 3 |
| **Legumes/vegetables** | 3827 | 0.80 | 0.81 | 0 | 4 |
| **Pasta** | 3827 | 0.51 | 0.69 | 0 | 4 |
| **Potato** | 3827 | 0.74 | 0.77 | 0 | 3 |
| **Meat** | 3827 | 1.07 | 0.83 | 0 | 5 |
| **Chocolate milk** | 3827 | 0.11 | 0.54 | 0 | 6 |

Table 2 – Loadings for each food item used in the principal components analysis. Only the first fives components are reported here, after varimax rotation. Children aged 12 months.

| ***Food item*** | ***Milk*** | ***Staple*** | ***Meat & vegetables*** | ***Beverage*** | ***Snack*** |
| --- | --- | --- | --- | --- | --- |
| **Breast milk** | **0.6825** | -0.0053 | -0.0518 | 0.0415 | 0.0385 |
| **Cow’s milk** | **-0.6955** | -0.0175 | -0.0444 | 0.0169 | 0.0033 |
| **Coffee** | 0.0564 | 0.0347 | 0.0847 | 0.0002 | **0.5343** |
| **Water/Tea** | -0.0014 | 0.0402 | 0.1331 | **0.7182** | -0.0475 |
| **Juice** | -0.0292 | 0.0583 | 0.1685 | **-0.6563** | -0.0283 |
| **Bread/Cookie** | 0.0157 | -0.0056 | 0.0529 | -0.0449 | **0.6268** |
| **Yogurt** | 0.1252 | -0.0761 | 0.0206 | -0.1886 | -0.2590 |
| **Fruit** | -0.0075 | 0.0770 | 0.2308 | -0.0047 | -0.3764 |
| **Egg** | -0.0048 | 0.0869 | -0.0473 | 0.0627 | -0.1838 |
| **Rice** | 0.0010 | **0.6543** | 0.1545 | 0.0015 | 0.0451 |
| **Beans** | 0.0081 | **0.5480** | -0.1066 | -0.0129 | -0.0278 |
| **Legumes/vegetables** | 0.0183 | -0.1701 | **0.5032** | 0.0262 | -0.1170 |
| **Pasta** | -0.0437 | **-0.4029** | 0.0653 | -0.0144 | 0.1402 |
| **Potato** | 0.0586 | -0.1784 | **0.3564** | 0.0361 | -0.0858 |
| **Meat** | -0.0204 | 0.1478 | **0.6779** | -0.0061 | 0.1186 |
| **Chocolate milk** | -0.1557 | 0.0273 | 0.0635 | 0.0863 | 0.1301 |

Figure 1 – Distribution of the scores obtained for the first five components of principal components analysis (scores multiplied by 100) and the screeplot. Children aged 12 months.

# Food profiles at 24 months of age

Table 3 – Sample size, mean, standard deviation, minimum and maximum for the frequency of consumption in 24 hours of each food item used for the profiles of children age 24 months.

| **Food item** | **N** | **Mean** | **s.d.** | **Min.** | **Max.** |
| --- | --- | --- | --- | --- | --- |
| **Breast milk** | 3790 | 0.63 | 1.35 | 0 | 7 |
| **Cow’s milk** | 3790 | 2.39 | 1.40 | 0 | 7 |
| **Coffee** | 3790 | 0.32 | 0.65 | 0 | 5 |
| **Water/Tea** | 3790 | 0.58 | 0.87 | 0 | 6 |
| **Juice** | 3790 | 1.15 | 1.09 | 0 | 6 |
| **Bread/Cookie** | 3790 | 1.33 | 0.84 | 0 | 5 |
| **Yogurt** | 3790 | 0.71 | 0.83 | 0 | 5 |
| **Fruit** | 3790 | 0.84 | 0.81 | 0 | 4 |
| **Egg** | 3790 | 0.15 | 0.40 | 0 | 2 |
| **Rice** | 3790 | 1.47 | 0.69 | 0 | 4 |
| **Beans** | 3790 | 1.27 | 0.78 | 0 | 4 |
| **Legumes/vegetables** | 3790 | 0.61 | 0.74 | 0 | 3 |
| **Pasta** | 3790 | 0.48 | 0.64 | 0 | 3 |
| **Potato** | 3790 | 0.56 | 0.70 | 0 | 3 |
| **Meat** | 3790 | 1.43 | 0.78 | 0 | 4 |
| **Chocolate milk** | 3790 | 0.59 | 1.14 | 0 | 6 |

Table 4 – Loadings for each food item used in the principal components analysis. Only the first fives components are reported here, after varimax rotation. Children aged 24 months.

| ***Food item*** | ***Staple*** | ***Milk*** | ***Snack*** | ***Beverage*** | ***Meat & vegetables*** |
| --- | --- | --- | --- | --- | --- |
| **Breast milk** | -0.0361 | **0.6496** | 0.0950 | -0.0510 | 0.0132 |
| **Cow’s milk** | -0.0246 | **-0.6883** | 0.0458 | -0.0231 | -0.0087 |
| **Coffee** | 0.0463 | 0.1155 | **0.5745** | 0.0213 | -0.0422 |
| **Water/Tea** | 0.0698 | 0.0293 | -0.0498 | **-0.7071** | 0.0729 |
| **Juice** | 0.0734 | 0.0176 | -0.0504 | **0.6841** | 0.0700 |
| **Bread/Cookie** | -0.0331 | 0.0225 | **0.5756** | 0.0204 | -0.0147 |
| **Yogurt** | 0.0097 | 0.2765 | **-0.4509** | 0.0810 | -0.0760 |
| **Fruit** | -0.0144 | -0.0152 | -0.1444 | 0.0833 | **0.4564** |
| **Egg** | 0.0857 | 0.0502 | -0.0677 | -0.0170 | -0.0880 |
| **Rice** | **0.6660** | -0.0068 | 0.0030 | -0.0156 | 0.0523 |
| **Beans** | **0.6057** | 0.0057 | -0.0016 | 0.0190 | -0.0584 |
| **Legumes/vegetables** | -0.0812 | 0.0265 | -0.0884 | -0.0837 | **0.5441** |
| **Pasta** | **-0.3806** | 0.0164 | -0.0108 | 0.0152 | -0.0077 |
| **Potato** | -0.0389 | -0.0201 | 0.1314 | 0.0505 | 0.3846 |
| **Meat** | 0.1141 | 0.0186 | 0.0944 | -0.0037 | **0.5605** |
| **Chocolate milk** | 0.0234 | -0.0906 | -0.2443 | -0.0618 | -0.0370 |

Figure 2 – Distribution of the scores obtained for the first five components of principal components analysis (scores multiplied by 100) and the screeplot. Children aged 24 months.

# Food profiles at 48 months of age

Table 5 – Sample size, mean, standard deviation, minimum and maximum for the frequency of consumption in 24 hours of each food item used for the profiles of children age 48 months.

| **Food item** | **N** | **Mean** | **s.d.** | **Min.** | **Max.** |
| --- | --- | --- | --- | --- | --- |
| **Breast milk** | 3714 | 0.06 | 0.36 | 0 | 5 |
| **Cow’s milk** | 3714 | 2.06 | 1.28 | 0 | 6 |
| **Coffee** | 3714 | 0.55 | 0.85 | 0 | 6 |
| **Water/Tea** | 3714 | 0.46 | 0.78 | 0 | 7 |
| **Juice** | 3714 | 1.00 | 1.01 | 0 | 7 |
| **Bread/Cookie** | 3714 | 1.42 | 0.88 | 0 | 5 |
| **Yogurt** | 3714 | 0.49 | 0.80 | 0 | 6 |
| **Fruit** | 3714 | 0.60 | 0.76 | 0 | 4 |
| **Egg** | 3714 | 0.15 | 0.40 | 0 | 3 |
| **Rice** | 3714 | 1.43 | 0.69 | 0 | 4 |
| **Beans** | 3714 | 1.14 | 0.80 | 0 | 4 |
| **Legumes/vegetables** | 3714 | 0.51 | 0.68 | 0 | 3 |
| **Pasta** | 3714 | 0.42 | 0.62 | 0 | 3 |
| **Potato** | 3714 | 0.48 | 0.66 | 0 | 3 |
| **Meat** | 3714 | 1.31 | 0.78 | 0 | 4 |
| **Chocolate milk** | 3714 | 1.44 | 1.35 | 0 | 5 |
| **Soft drink** | 3714 | 0.59 | 0.88 | 0 | 5 |
| **Chips** | 3714 | 0.39 | 0.40 | 0 | 6 |
| **Sweets** | 3714 | 1.91 | 2.06 | 0 | 7 |
| **Chocolate** | 3714 | 0.33 | 0.46 | 0 | 7 |

Table 6 – Loadings for each food item used in the principal components analysis. Only the first fives components are reported here, after varimax rotation. Children aged 48 months.

| ***Food item*** | ***Staple*** | ***Milk*** | ***Snack*** | ***Beverages*** | ***Treats*** |
| --- | --- | --- | --- | --- | --- |
| **Breast milk** | -0.0257 | -0.1170 | 0.0314 | 0.0064 | 0.0981 |
| **Cow’s milk** | -0.0064 | **0.6382** | -0.0295 | -0.0150 | 0.0115 |
| **Coffee** | 0.0275 | -0.2768 | **-0.4613** | -0.0302 | 0.1037 |
| **Water/tea** | 0.1302 | 0.0028 | **-0.3402** | -0.2597 | -0.2472 |
| **Juice** | 0.0141 | -0.0458 | 0.1025 | **0.7140** | 0.0569 |
| **Bread/Cookie** | -0.0364 | -0.1464 | **-0.3453** | 0.0393 | 0.1018 |
| **Yogurt** | -0.0697 | -0.2561 | 0.4177 | 0.0904 | -0.0637 |
| **Fruit** | 0.1220 | -0.0306 | 0.1860 | 0.2010 | -0.1416 |
| **Egg** | 0.0063 | 0.0392 | -0.1450 | -0.0065 | 0.0367 |
| **Rice** | **0.6212** | -0.0095 | -0.0291 | -0.0064 | 0.0315 |
| **Beans** | **0.5029** | 0.0324 | -0.1597 | 0.1076 | 0.0331 |
| **Legumes/vegetables** | 0.2214 | 0.0158 | 0.1411 | 0.1403 | -0.1044 |
| **Pasta** | -0.2327 | -0.0489 | -0.0311 | 0.1839 | 0.0399 |
| **Potato** | 0.1884 | -0.0696 | 0.1931 | -0.1636 | 0.0172 |
| **Meat** | **0.4225** | -0.0564 | 0.2181 | -0.0369 | 0.0132 |
| **Chocolate milk** | -0.0044 | **0.6217** | 0.0479 | 0.0086 | 0.0260 |
| **Soft drinks** | -0.0313 | -0.1021 | **0.3870** | **-0.5247** | 0.1466 |
| **Chips** | 0.0226 | 0.0210 | -0.0116 | -0.0205 | **0.5784** |
| **Sweets** | 0.0517 | 0.0051 | -0.0820 | 0.0052 | **0.5650** |
| **Chocolate** | -0.0063 | 0.0403 | 0.1374 | 0.0489 | **0.4339** |

Figure 3 – Distribution of the scores obtained for the first five components of principal components analysis (scores multiplied by 100) and the screeplot. Children aged 48 months.
